# Relation between Double Layer Structure, Capacitance, and Surface Tension in Electrowetting of Graphene and Aqueous Electrolytes

**DOI:** 10.1021/jacs.3c10814

**Published:** 2023-12-28

**Authors:** Zixuan Wei, Joshua D. Elliott, Athanasios A. Papaderakis, Robert A.W. Dryfe, Paola Carbone

**Affiliations:** † Department of Chemical Engineering, The University of Manchester, Oxford Road, Manchester M13 9PL, United Kingdom; ‡ Diamond Light Source, Diamond House, Harwell Science and Innovation Park, Oxfordshire, Didcot OX11 ODE, United Kingdom; § Department of Chemistry and Henry Royce Institute, The University of Manchester, Oxford Road, Manchester M13 9PL, United Kingdom

## Abstract

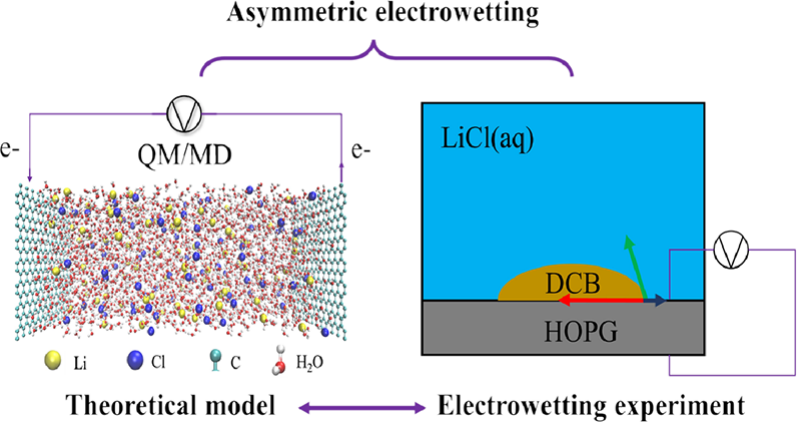

Deciphering the mechanisms of charge storage on carbon-based materials is pivotal for the development of next-generation electrochemical energy storage systems. Graphene, the building block of graphitic electrodes, is an ideal model for probing such processes on a fundamental level. Herein, we investigate the thermodynamics of the graphene/aqueous electrolyte interface by utilizing a multiscale quantum mechanics–classical molecular dynamics (QM/MD) approach to provide insights into the effect of alkali metal ion (Li^+^) concentration on the interfacial tension (γ_*SL*_) of the charged graphene/electrolyte interface. We demonstrate that the dependence of γ_*SL*_ on the applied surface charge exhibits an asymmetric behavior relative to the neutral surface. At the positively charged graphene sheet, the electrowetting response is amplified by electrolyte concentration, resulting in a strongly hydrophilic surface. On the contrary, at negative potential bias, γ_*SL*_ shows a weaker response to the charging of the electrode. Changes in γ_*SL*_ greatly affect the total areal capacitance predicted by the Young–Lippmann equation but have a negligible impact on the simulated total areal capacitance, indicating that the EDL structure is not directly correlated with the wettability of the surface and different interfacial mechanisms drive the two phenomena. The proposed model is validated experimentally by studying the electrowetting response of highly oriented pyrolytic graphite over a wide range of electrolyte concentrations. Our work presents the first combined theoretical and experimental study on electrowetting using carbon surfaces, introducing new conceptual routes for the investigation of wetting phenomena under potential bias.

## 1 Introduction

The development of robust electrochemical energy storage devices exhibiting fast charge storage kinetics and high Coulombic efficiency over multiple operation cycles is an ongoing technological challenge. Aqueous electrical double-layer capacitors (supercapacitors) using carbon-based materials are considered to be a highly promising and environmentally sustainable option for the energy storage grid.^1−3^ However, the energy density of traditional supercapacitors is low due to the mechanism of the overall charge storage process, which involves the reversible non-Faradaic physisorption of ions inside the electrical double layer (EDL).^4^ Enhancing the capacitance of supercapacitors is therefore crucial to improving the energy density of such devices. Graphene^5−11^ possesses a high surface area (2630 m^2^ g^–1^ for monolayer graphene) compared to amorphous carbon that can be utilized for the accumulation of ions within the EDL and thus significantly increase the capacitance of the electrodes. However, the successful utilization of the electrochemically active surface area of the electrode is directly related to its accessibility by the electrolyte (e.g., ingress of the electrolyte into its porous structure). On this basis, understanding the mechanism of wetting at the carbon/aqueous electrolyte interface under potential bias (the phenomenon of electrowetting^12^) is essential in the pursuit of strategies to enhance the capacitance of the devices.^13−15^

Electrowetting is an electrocapillary phenomenon that was identified initially by Lippmann,^16^ who demonstrated that the position of the mercury meniscus in a capillary can be controlled by applying an external voltage. Nowadays, the term electrowetting is generally referred to any situation where the application of voltage induces shape variations of a liquid/liquid or liquid/gas interface.^17,18^ Experiments are normally performed with a dielectric layer between the electrode and the solution (electrowetting on dielectric, EWOD) in order to prevent the degradation of both the substrate and the electrolyte (i.e., electrolysis). In this case, the applied potential difference charges both sides of the dielectric layer so that the capacitance of the system is set by the thickness of the dielectric layer, and large voltages are needed to induce changes in surface energy and hence wetting. An alternative strategy that tackles the high energy demands of EWOD is the use of ultrasmooth conductive substrates, which, together with an accurate tuning of the underlying physicochemical processes, allows electrowetting experiments to be performed without the need of the dielectric layer.^19,20^ This approach is referred to as electrowetting on conductors (EWOC).^21^

Graphene, with its atomically smooth surface area and unique physicochemical properties can be potentially used as an electrode for EWOC, allowing the construction of ultralow voltage “electrowetting on conductor” devices that could modify the surface properties upon the applications of a small voltage.^22,23^ The electrowetting phenomenon is conventionally described at the macroscopic level by the Young–Lippmann equation (eq 1), which predicts that upon application of a voltage the liquid–solid contact angle (θ) decreases from its equilibrium value (θ_0_) at zero surface charge, depending on the magnitude of the applied voltage (*V*), the total areal capacitance (*C*_*YL*_), and the liquid-ambient (liquid–air) surface tension (γ_*LV*_).
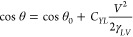
1

The Young–Lippmann equation predicts a constant decrease of the contact angle as voltage increases until the surface is completely wet, and a symmetric response relative to the neutral surface for positive and negative voltages.^24^ Experiments performed with different dielectric layers show, however, that the value of the contact angle quickly saturates as the voltage increases and the liquid never fully spreads. This mismatch between theoretical predictions and experimental observations has been explained in various ways including the need to account for the electric field distribution at the triple contact line (e.g., solid/liquid/air) and for the balance between the binding force among the liquid molecules and the drop spreading in reducing the free energy of the system.^25,26^ Molecular simulations have been used in an attempt to explain the reasons behind the inability of the Young–Lippmann equation to predict the saturation effect. At present, there are two major hypotheses: one is the electromechanical explanation,^26^ which suggests that the application of high voltages leads to the electric force at the drop’s edge exceeding the molecular binding force resulting in molecules being pulled from the drop and to saturation. Another hypothesis is purely thermodynamic and suggests that the main contributor to the reduction of the contact angle is a reduction in the solid–liquid interfacial tension γ_*SL*_^12,24^ that happens upon charging the surface. As the EDL layer spontaneously builds up at the liquid–solid interface, charges of opposite polarity are repelled from the interface, thus leading to a decrease in the γ_*SL*_ and consequently a reduced equilibrium contact angle, making the surface more hydrophilic.^27^ Rewriting eq 1 in terms of surface tension, the Young–Lippmann equation becomes^28^
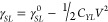
2where γ_*SL*_^0^ is the solid/liquid interfacial tension at the neutral surface. Molecular simulations assume that the wetting occurs directly on the conductor surface; however, as previously mentioned, most of the experiments are conducted with a dielectric placed between the electrode and the electrolyte. In this case, the electrostatic energy is mainly stored in the dielectric layer, rather than in the EDL.^29^ Papaderakis et al.^19^ have recently described EWOC experiments using the basal plane of atomically smooth graphite (highly oriented pyrolytic graphite, HOPG) in direct contact with aqueous electrolytes of alkali metal halides of increasing ionic concentrations up to the “water-in-salt” range. Their findings demonstrate that a decrease in the electrolyte concentration results in a strongly asymmetric contact angle dependence on the applied voltage relative to the potential of zero charge. They interpreted that the observed asymmetry on the basis of the underlying electrochemically induced charge transfer processes occurring at the surface of graphite and their effect on the surface charge. Their contact angle data also indicate that the Young–Lippmann model holds for highly concentrated electrolytes due to the expansion of the purely capacitive region (i.e., the voltage range within which no charge transfer reactions occur) as a consequence of the suppression of the underlying parasitic reactions. On the contrary, at concentrations below the water-in-salt regime, significant deviations from the Young–Lippmann equation are recorded. However, it is difficult to experimentally observe the changes in EDL structure with electrolyte concentration and applied voltage, which are key elements that drive electrowetting at the interface.

To quantify how these changes drive electrowetting, herein we directly calculate, using molecular dynamics, the solid/liquid surface tension (γ_*SL*_) of LiCl electrolyte solutions of different concentrations in contact with a neutral and charged graphene sheet. To account for the polarization effect induced on the electrode surface by the presence of an external voltage and the electrolyte, we employ a novel procedure that combines the explicit polarization of the graphene electrodes, modeled at the quantum mechanical (QM) level, with the long time scale motion of the electrolytes in the solution modeled via molecular dynamics (MD). The procedure, called QM/MD, balances accuracy and computational cost and has been already used to model graphene/electrolyte interfaces^30,31^ and also conducting polymer solutions.^32^ The change in γ_*SL*_ upon charging the graphene electrodes is calculated by integrating pressure differences along the direction perpendicular to the graphene surface (*z*) between the pressure normal (*P*_*N*_) and that tangential (*P*_*T*_) to the graphene surface as obtained from the MD simulations^33^
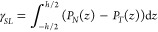
3where *h* is the distance from the electrode surface to the bulk electrolyte. This method has been proven to be applicable on solid/liquid interfaces.^34−37^ The simulations allow also for the direct evaluation of EDL capacitance (*C*_*YL*_ in equation 2) providing insight into the applicability of the Young–Lipmann theory and the effect of γ_*SL*_on the charge storage mechanism at the interface. The simulation results are then compared with experimental contact angle and capacitance data obtained from electrowetting experiments carried out on a HOPG within the same voltage windows used in the simulations. The choice of HOPG as a model surface relies on the fact that graphene samples produced by chemical vapor deposition techniques are intrinsically defective and often contain impurities (such as residual polymer particles) introduced during the transfer process. Both phenomena have been found to strongly influence the electrowetting response.^22,23,38^ Furthermore, the electronic interactions between the graphene overlayer and the substrate are expected to affect the wetting response. Such phenomena have not been considered in our calculations and therefore a direct comparison would be rather ambiguous.^36,38^ On this basis, though we recognize that the wetting behavior of the two pristine surfaces (i.e., defect-free, uncontaminated, free-standing graphene, and highly oriented graphite) can differ, we believe that our approach can effectively provide significant insights into the wetting behavior of carbon surfaces under external potential bias by linking theory and experiments. In this respect, we envision that the reported data and approaches assist the development of models that encompass the complete physics of the carbon/aqueous electrolytes interface. Finally, we further note that in order to minimize the experimental uncertainties often appearing in electrowetting experiments when using the traditional liquid/air configuration (related mainly to the environmental effects, i.e., airborne contaminations, electrolyte evaporation, and the necessary use of a micropipette to establish an electrolytic contact between the drop and the substrate^19^), we adopted the pioneering liquid/liquid configuration introduced by Frumkin,^21,39^ where an electrolytically inactive solvent is used in combination with the electrolyte of interest (refer to the next section for a detailed description of the mechanism of the process).

## 2 Materials and Methods

### 2.1 Computational Methods and Models

**Figure 1:**
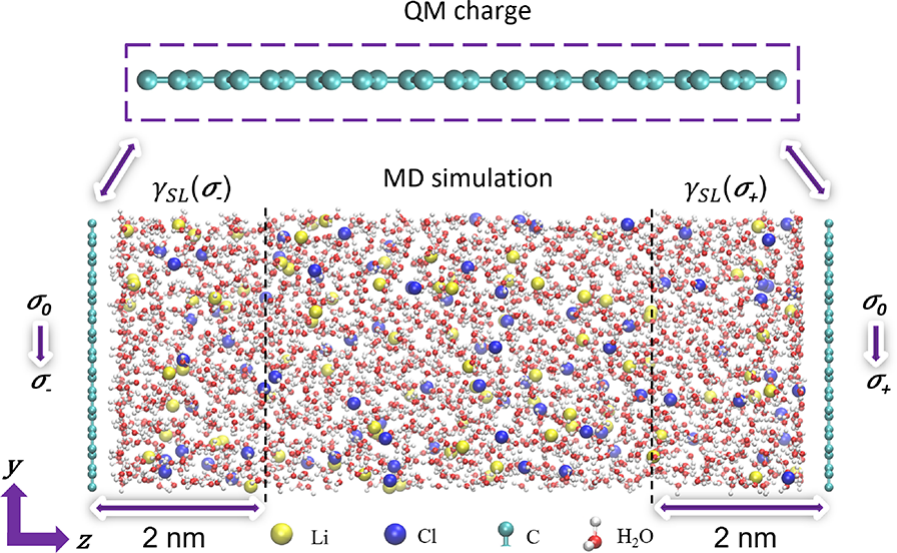
Aqueous graphene-based supercapacitor model. The left/right polarizable graphene electrodes are either neutral (σ_0_= 0.000 *Cm*^2–^) or negatively/positively charged (σ_–_/σ_+_ = – 0.071/0.071 *Cm*^2–^). Atomic charges are obtained at the SCC-DFTB level of theory. The polarizable electrodes are in contact with a 2 M LiCl solution that is time-evolved using classical molecular dynamics (MD). γ_SL_(σ_–_) and γ_SL_(σ_+_) are the electrode–electrolyte interfacial tensions, which are calculated as the integral of the pressure difference from the electrode surface to the bulk layer. Dashed black lines represent the position where the bulk phase is obtained.

Figure 1 presents an aqueous graphene-based supercapacitor model consisting of two infinitely rigid graphene electrodes (3 × 3 nm) in contact with the 8 nm slab of LiCl solution. We also include a region of vacuum 16 nm thick in nonperiodic direction parallel to the electrode surface normal in order to reduce any long-ranged interactions between periodic images.^40−42^ To study the impact of different LiCl concentrations and electrode surface charge densities on electrowetting, we created models with three fixed surface charge densities: (i) a neutral (σ_0_ = 0.000 C m^–2^) case, (ii) a negatively charged case, and (iii) a positively charged (σ_–_ = – 0.071 C m^–2^ and σ_+_ = + 0.071 C m^–2^) case. The choice of the surface charge was based on our previous work^30,31,43^ that showed that such values guarantee the resulting electrostatic potential to be within the limits of stability set by the reduction/oxidation of water.^31^ The ionic concentrations in these models range from 0 to 4 M. Periodic boundary conditions are applied for all models.

Before starting the QM/MD simulation, graphene structure was optimized at the PBE-DFT^44,45^ level using the Quantum Espresso software suite^46,47^ and Optimized Norm Conserving Vanderbilt pseudopotentials.^48^ The optimization resulted in a carbon-carbon bond length of 0.143 nm. The QM/MD simulation was performed using the iterative process developed by Elliott et al.^30^ with more details in Figure S1 in the SI, which combines quantum mechanical self-consistent charge (SCC) density functional tight-binding (DFTB) calculations of the electrode’s electronic structure with classical molecular dynamics trajectories of the electrolyte. This approach allows for the capture of the dynamic and structural evolution of the electrode/electrolyte interface during the process of electrolyte-induced polarization of the surface.

In our previous work,^30,31^ in order to model the graphene–electrolyte interface, we adopted a simple QM/MD scheme consisting of one electrode in contact with the electrolyte. In two independent simulations, this electrode was then charged positively and negatively to capture the behavior of the anode and cathode. However, this setup increases the computational cost, as two independent simulations are necessary. Additionally, this places further constraints on the simulations that may be carried out. In order to preserve the overall net charge neutrality, the composition of the electrolyte must be augmented with additional ions to counter balance the charge of the electrode. Moreover, to avoid further complications, this means that the total electrode charge can only be incremented in integer multiples of the counterionic charge. To this end, here, we have implemented a two-electrode QM/MD setup that alleviates all of these constraints and enables us to simultaneously sample the interface at the anode and cathode. From a practical standpoint, the procedure for the two-electrode setup is analogous to that of the single electrode case in our previous work. In two separate QM calculations, the anode and cathode electronic structures are computed in the presence of the background electrostatic potential of the electrolyte atoms. This yields a set of atomic charges (computed via Mulliken population analysis) for each electrode. These atomic charges are mapped onto the classical MD force field and used for the time evolution of the electrolyte. After a fixed time step of 5 ps, the QM charges on the electrode are re-evaluated. The QM/MD simulation of each model is carried out for 160 ns, with the first 10 ns dedicated to system equilibration.

For QM calculations, the DFTB+ software package is employed, which computes the electronic structure at the SCC-DFTB level.^49^ For this class of systems, we previously found that a relatively loose set of simulation parameters may be used to obtain satisfactorily converged atomic charges. This helps to keep the cost of the QM part of the simulation at a minimum. To this end, we sample the first Brillouin zone at the Γ point only and we apply a Fermi temperature of 1 × 10^–6^ K for smearing the occupancies of the electronic states. The convergence threshold of the self-consistent charge optimization is set to 1 × 10^–2^ Hartree. In the MD simulation, we run the model under NVT ensemble using GROMACS (version 2018.4).^50,51^ The Nosé–Hoover thermostat is employed fixing the temperature of 298 K with relaxation times of 1 ps. The cutoff distance of short-range Lennard-Jones and electrostatic interactions is set to 1.4 nm. The long-range electrostatic interaction is treated by the particle Mesh ewald approach with standard (3D) geometry. The switch function of the Lennard Jones 12–6 potential is onset from 1 nm to smoothly truncate at 1.4 nm. The TIP4P-2005^52^ is used as the water model with the rigid constraint by SETTLE algorithm. The parameters of ions (Li^+^ and Cl^–^) and carbon atoms are taken from the Madrid-2019 force field^53^ and the Amber force-field,^54^ respectively. The Lorentz–Berthelot combining rule is used for the nonbonded ion-carbon and oxygen-carbon interactions. Graphene electrodes are frozen during the simulation. The outputted trajectories undergo postprocessing using GROMACS-LS (version 2016.3)^55^ to obtain local stress for calculating γ_*SL*_. The averaged γ_*SL*_ is calculated by taking the mean of the sum of sub γ_*SL*_ calculated in each iteration of the QM/MD loop.

### 2.2 Experimental Section

Highly oriented pyrolytic graphite (HOPG; ZYA quality, mosaic spread 0.4 ± 0.1 ° supplied by Scanwel, UK) served as the working electrode (WE). Electrical connection was made by stripping an enamel copper wire (RS components, UK) for about 0.5 cm at each end and attaching one side to the edge plane of the HOPG with conductive silver epoxy resin (RS component, UK). Following overnight curing, the epoxy was covered with an insulating resin and left to dry for a few hours. In this way, the direct contact of the silver epoxy and stripped copper wire end with the electrolyte (and hence the introduction of ionic impurities in the latter) was eliminated. A platinum mesh was used as a counter electrode (CE) and a custom-made Ag/AgCl_(3 M KCl)_ electrode with an agarose frit as a reference electrode (RE). Details about the fabrication procedure of the RE can be found in Papaderakis et al.^56^ Unless otherwise specified, the potential in the experimental data is referred vs Ag/AgCl_(3 M KCl)_. The electrolyte solutions were freshly prepared within the reported LiCl (BioXtra ≥ 99% from Sigma Aldrich) concentration range and purged with nitrogen prior to use. For the insulating (no added electrolyte) droplet, small amounts (within a few μL range) of 1,2–dichlorobenzene (DCB, 99% from Sigma Aldrich) were used.

**Figure 2:**
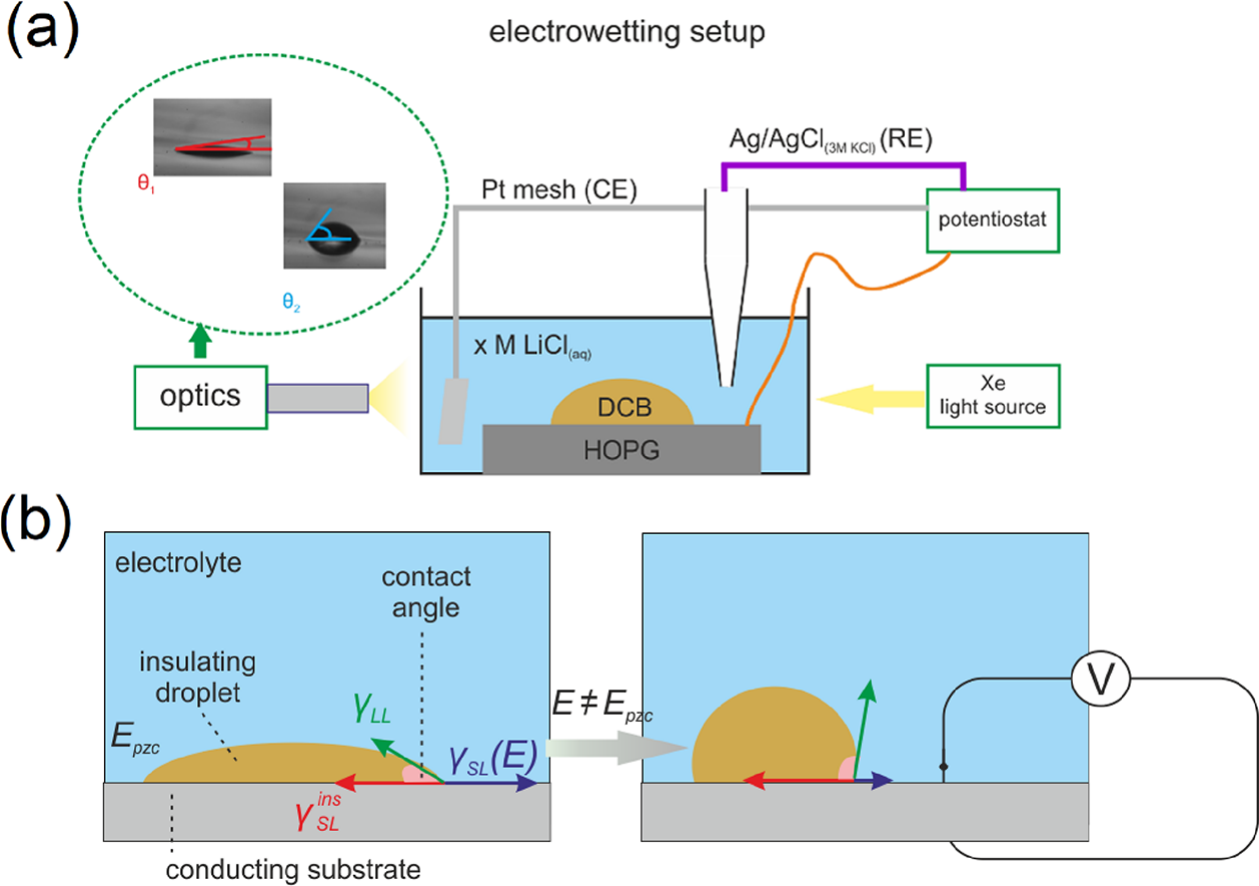
(a) Schematic representation of the experimental setup used for the electrowetting experiments. (b) Illustration of the mechanism of electrowetting at the electrode/electrolyte/insulator interface. At the potential of zero charge (*E_pzc_*), the electrode/electrolyte interfacial surface tension γ*_SL_*(*E*) obtains its maximum value, and hence, the insulating droplet exhibits its lowest contact angle. Upon the application of a potential bias, the potential dependent γ_SL_(E) decreases, while the potential independent γ*_LL_* and γ*_SL_^ins^* (representing the interfacial surface tensions at the electrolyte/insulator and electrode/insulator interfaces, respectively) remain unchanged. This phenomenon drives the receding motion of the droplet depicted in the increase in its contact angle. The larger the extent of decrease in γ*_SL_*(*E*), the higher the equilibrium contact angle of the droplet.

A sketch of the setup used for the electrowetting experiments is illustrated in Figure 2a. HOPG was freshly cleaved and placed at the bottom of a quartz container. Within a few seconds, the container was filled with the surrounding aqueous electrolyte (LiCl) to minimize the adsorption of airborne impurities on the surface of the electrode. Subsequently, the DCB droplet was deposited on the basal plane of HOPG by the controlled flow of the solvent through a micropipette using a microinjector (PV820 Pneumatic PicoPump, from World Precision Instruments). The micropipette was fabricated by pulling a borosilicate glass capillary (inner diameter 0.84 mm, outer diameter 1.5 mm, length 10.16 cm, from World Precision Instruments) with a Sutter P-97 Flamming/Brown micropipette puller. The resultant tip diameter was in the range of 5–6 μm, and the diameter of the DCB droplet was in the range of 300–500 μm. Manual micropositioners (Thor Laboratories) were used to control the position of the substrate and the pipet. After droplet deposition, the micropipette was retracted from the surface, and the CE and RE were carefully placed in the quartz container.

The shape variations of the droplet with the applied potential bias were monitored using a Photron FASTCAM SA3 high speed camera controlled via a Photron FASTCAM Viewer and a Storz Xenon Nova 300 light source. The recorded image captions under static conditions (i.e., after an equilibrium contact angle was achieved; within tenths to hundreds of ms) were analyzed using a custom-made Canny edge image processing algorithm in Matlab, Math-Works Inc., Natick MA, USA (for details, see Papaderakis et al.^19^) to determine the equilibrium contact angle at each applied potential bias.

Electrowetting measurements were performed by using an Autolab PGSTAT302N potentiostat (Metrohm) operated via Nova 1.11.2 software. The experimental procedure followed for monitoring the contact angle dependence on the applied bias comprised consecutive potential pulses from 0 V to either – 1.5 or + 1.3 V with a step of 50 mV. The duration of the pulses was adjusted to 10 s.

The mechanism of electrowetting in the liquid/liquid configuration adopted for the experiments is illustrated in Figure 2b, where γ_*SL*_(*E*), γ_*SL*_^*ins*^, and γ_*LL*_ are the solid/electrolyte, solid/insulator (solvent), and electrolyte/insulator interfacial surface tensions, respectively. Note that within the applied potential range only γ*_SL_*(*E*) is potential-dependent. This is due to the fact that in contrast to the solid/electrolyte interface where the potential drop extends within a range defined by the EDL thickness (i.e., on the order of the Debye length of the electrolyte), the corresponding potential drop at the droplet/electrode interface is distributed across the entire microscopic droplet. The latter would necessitate the application of a significantly higher energy input to overcome the introduced ohmic drop, which would far exceed the potential range employed in this study. Initially, the insulating droplet rests at the potential of zero charge (*E*_*pzc*_) where it exhibits its minimum contact angle (also at *E*_pzc_, γ_*SL*_(*E*) obtains its maximum value, conceptually similar to the maximum observed at *E*_*pzc*_ in the classical electrocapillary curves). Upon application of a bias away from *E*_*pzc*_, the decrease in γ_*SL*_(*E*) drives the receding motion of the insulating droplet and therefore the increase in its contact angle.^19,21^ The overall process is macroscopically described by combining Young’s equation with the thermodynamic definition of EDL capacitance at a constant chemical potential, μ_i_, as follows:
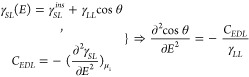
4

In the above relation, the potential drop at the interface is expressed as the voltage difference, *E*, between the working and reference electrodes, both being immersed in the same solution, in which the latter has a well-defined electrochemical potential. Equation 4 can be easily rearranged into the Young–Lippmann equation (eq 1), where the negative sign is eliminated due to the larger θ values at *E* ≠ *E*_pzc_ arising from the decrease in γ*_SL_*(*E*) away from *E*_pzc_.

With this approach, initially introduced by Frumkin,^39^ the changes in the electrode/electrolyte interfacial surface tension can be accurately investigated. In other words, a decrease in the electrode/electrolyte interfacial surface tension (or a decrease in contact angle in terms of an electrode in contact with air) will be reflected in an increase in the contact angle of the insulating droplet.

## 3 Results and Discussion

### 3.1 Structure of Electrode/Electrolyte Interface

**Figure 3:**
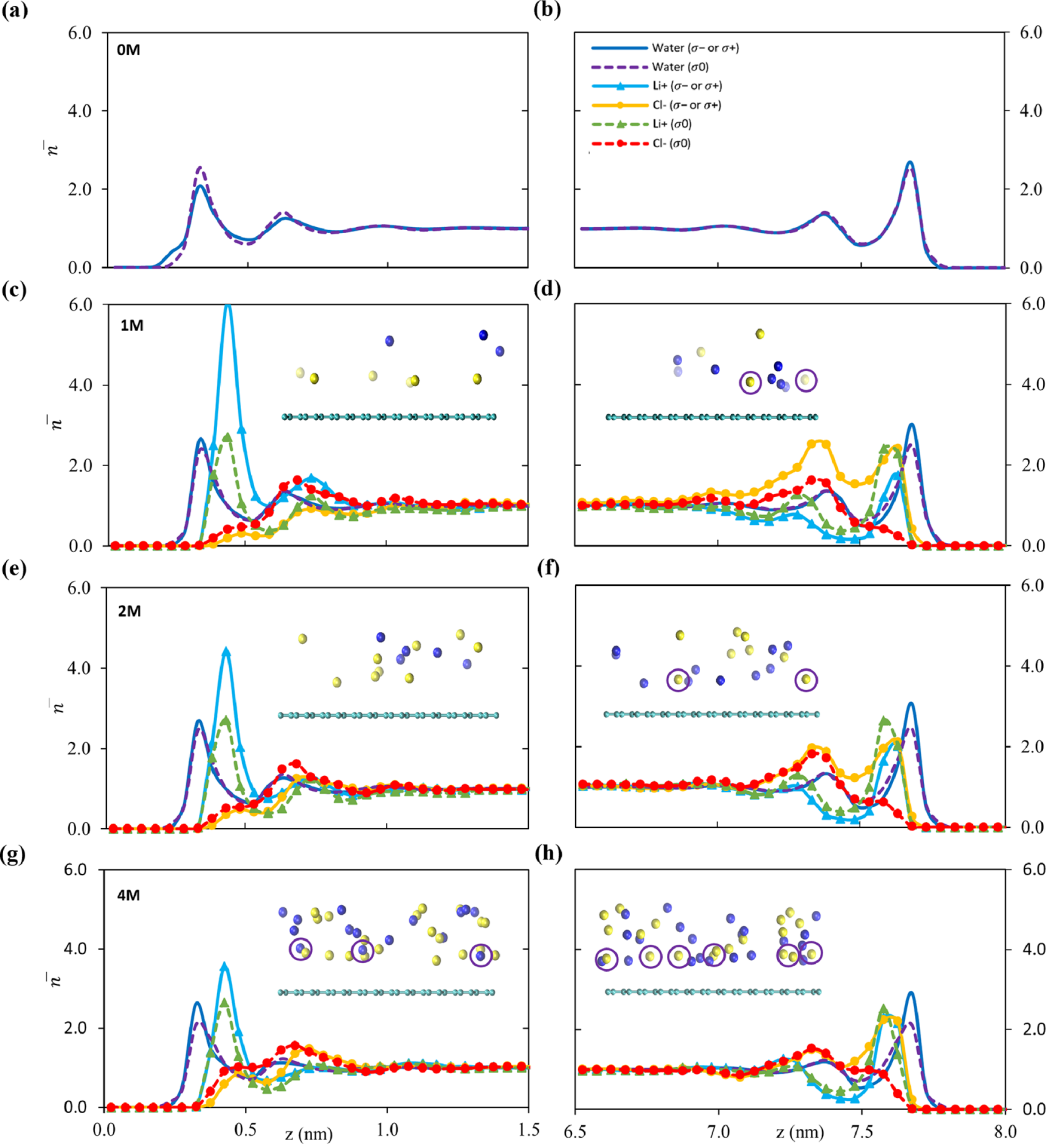
Bulk-normalized number density, *n̅*(*z*), of water (dark blue solid line), Li^+^ (blue solid line with triangle), and Cl^–^(yellow solid line with circle) at the negative (left) and positive (right) electrodes. Purple dashed line, green dashed line with triangle, and red dashed line with circle correspond to water and Li^+^ and Cl^–^ density profiles in the case that both electrodes are overall charge neutral. The graphene electrodes are placed at positions of *z* = 0.0 nm (cathode) and 8.0 nm (anode). From top to bottom, panels represent 0, 1, 2, and 4 M concentrations of LiCl, respectively. Inside snapshots of ions concentrating at the charged surface (σ_–_ and σ_+_) for different concentrations, ions in purple circle are in the OHP, and they contribute to the overscreening effect.

We start analyzing the changes in the electrode/electrolyte interfacial structure by calculating the bulk-normalized number density profile *n̅*(*z*) along the *z* axis, as a function of electrolyte concentration and surface charge
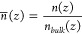
5where *n*_*bulk*_(*z*) represents the number density of given species in the bulk region. The *n̅*(*z*) for each given species is shown in Figure 3.

Throughout all systems, two distinct water peaks are consistently observed adjacent to the interface at 0.33 and 0.63 nm, regardless of the surface charge density. This characteristic positioning of the water peaks aligns with previous findings in the system of graphene and TIP4P/2005 water.^34,36^

In the system of pure water shown in Figure 3a,b, it is observed that upon charging the electrode negatively, the water molecules get closer to the electrode surface compared to the neutral case, while no changes in the water density profile are observed when the electrode is positively charged. This difference is due to different water orientation toward the surface occurring upon surface charging. In the positively charged cases, the water molecules are always oriented with the oxygen atom facing the surface, while as the potential is switched to negative, the water molecules flip and the hydrogen atoms face the surface instead (Figure 5c,d). Thus, the additional peak closer to the graphene shown in the density profile of Figure 3a on the cathode (i.e., negatively charged graphene), corresponds to the hydrogen atoms (Figure S2 in the SI). It is interesting to notice that this flip in orientation occurs also in the LiCl solutions for all concentrations (Figure 5c,d). Similar layering of water, which survives also at high concentration of salt,^58^ has been observed also in charged pores and has been associated with a change in surface wettability.^59^ Simulations have shown that when an externally applied electric field forces the alignment of the water molecules with the oxygen atoms facing the surface, its wettability is strongly enhanced. In line with these results, as we present later, our simulations indicate that positively charged graphene surfaces wetted by water are more hydrophilic than negative and neutral ones.

A similar normalized density profile is also calculated for the ions (Figure 3c–h). We observe that the Cl^–^ anions are always repelled from the neutral surface, regardless of the ionic concentration, in agreement with our previous simulation results.^31^ On the other hand, the Li^+^ cations always exhibit two adsorption peaks at 0.42 and 0.72 nm from the neutral surface, both outside the inner Helmholtz plane (IHP) that extend no further than a monolayer of water molecules along the *z*-axis from the graphene surface.^31^ The position of these peaks does not change even for highly concentrated solutions due to the relatively high hydration free energy^60^ of Li^+^ that prevents partial dehydration of its first hydration sphere. The position of the Li^+^ adsorption peaks remain unchanged, also when the electrode is negatively charged, but the first peak at 0.42 nm increases in height (Figure S3 in the SI). This is a result of the tradeoff between the dehydration free energy and the surface/ Li^+^ coulomb attractive interactions which, even if further enhanced by the surface polarization, never exceeds the former.^31,43,61^ As observed for Li^+^, the increase in electrolyte concentration increases only the amount of Cl^–^ near the positively charged electrode, while the position of the peaks is not affected by the concentration changes. However, at a 4 M concentration, this is accompanied by broadening of the second peak (Figure S3). The density profiles highlight an important feature of the double layer structure formed at the interface. Both anions and cations lie within the outer Helmholtz plane (OHP), extending no further than the first ionic adsorption layer along the *z*-axis from the graphene surface, and as the electrolyte concentration increases from 1 to 2 M, the density peaks clearly overlap (also see inside snapshots in Figure 3d,f). The coexistence of ions and counterions inside the Helmholtz plane, also evident in the non-normalized density profiles (Figure S3), suggests an overscreening effect, where the first adsorbed layer of ions provides higher counter charge than that on the electrode surface. The latter is compensated for by the adsorption of counterions that reside in between/in the vicinity of the first layer of ions. The overall phenomenon is evident on the positive electrode already at 1 M and is further amplified as concentration increases (also see inside snapshots in Figure 3d,f,h). At the negative surface, this overscreening is only visible for the 4 M concentration (also see inside snapshot in Figure 3g) and is significantly weaker when compared to the positive electrode. Overscreening effects and ion crowding phenomena in the EDL have been also predicted by Finney et al. in aqueous NaCl electrolytes on graphite at concentrations greater than ca. 0.6 M.^62^ Furthermore, similar effects have been reported in the literature for pure ionic liquids at relatively low applied voltages.^63^ All details of placement and thickness of IHP and OHP are reported in Figure S3 and Table S1, respectively.

### 3.2 Electrode/Electrolyte Interfacial Tension

**Figure 4:**
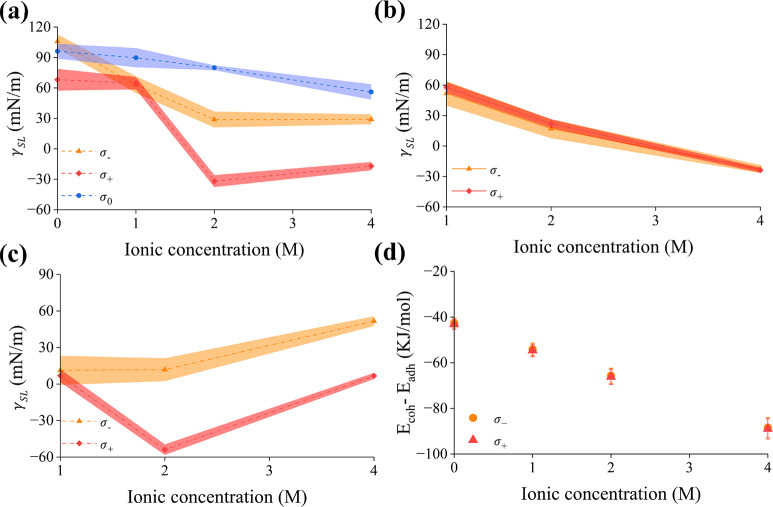
(a) Values of γ*_SL_* for different charged surfaces (σ_0_, σ_–_, and σ_+_) with their standard deviation (shaded error bands) as the function of the ionic concentration. (b–c) Contribution of the water molecules and ions to the γ_*SL*_ as a function of concentration and electrode surface charge with the standard deviation (shaded error bands). (d) Difference between cohesion and adhesion energy for different charged surfaces (σ_–_ and σ_+_) as a function of the ionic concentration. The cohesion energy is calculated using all the water and ions and the adhesion energy using only the interfacial water and ions. The cohesive and adhesive energies have been normalized by their corresponding number of water and ions.

Figure 4a plots the electrode/electrolyte interfacial tension (γ_*SL*_) for pure water and the three ionic concentrations as a function of the applied surface charged densities. The results are also reported in Table S2, and all the *P*_*N*_(*z*) – *P*_*T*_(*z*) values with the corresponding running integral value of γ_*SL*_ along *z* (equation 3) are provided in Figure S4 for the different surface charge densities and ionic concentrations. For pure water, γ_*SL*_ at the neutral surface is found to be 96.2 ± 7.3 mN/m, consistent with previous studies on graphene/water interfacial tension.^34,36^ It is noteworthy that this value is consistent with that calculated by us using the same force field combination but with a model that does not account for the surface polarization,^36^ indicating that this does not affect the surface tension value when the surface is uncharged. Upon positively charging the electrode surface, γ_*SL*_ significantly decreases, while it remains unchanged at negative polarization. The calculated dependence of γ_*SL*_ on the applied surface charge relative to its value at the neutral surface demonstrates the asymmetric nature of electrowetting. Calculating the static contact angle using classical molecular dynamics simulations, Taherian et al.^28^ and Daub et al.^64^ also observed that pure water prefers to wet positively charged graphene surfaces rather than negative ones. Taherian et al.^28^ observed asymmetric contact angles also for a 4 M NaCl solution and a room temperature ionic liquid (1-butyl-3-methylimidazolium tetrafluoroborate). Despite finding a larger reduction in the contact angle for positively charged surfaces, the authors did not find the same asymmetric behavior when calculating the solid/liquid surface tension. The disagreement with the Young equation was attributed to the large influence of the water molecular orientation in the direct vicinity of the contact line, which might be dominant when, as in their case, the applied surface charge density is very small. Our QM/MD simulations indicate, however, that, at higher surface charge density (although still experimentally relevant), the observed reduction of contact angle goes hand in hand with a reduction in surface tension as expected by the Young equation.

As the electrolyte is added, the asymmetry in the electrowetting response either disappears (1 M) or is somewhat reduced (i.e., for 2 and 4 M, the surfaces become hydrophilic irrespective of their charge, although the positive electrode is still more hydrophilic than the negative one). As soon as the surface is charged (either negatively or positively), the surface tension drops by around 30%. For low concentration (1 M), the anode and cathode are equally wettable as we show below: at this concentration, the effect of the adsorbed Cl^–^ anions on the surface tension is negligible and the electrowetting effect is dominated by water. For higher concentrations (2 and 4 M), we find that the negatively charged surface is more hydrophilic than the neutral surface. For the positively charged surface, the value of γ_*SL*_ becomes negative, which indicates that the electrode surface is completely wet. In general, γ_*SL*_ decreases as the concentration of LiCl increases. This phenomenon starts to become evident for concentrations above 2 M, where the γ_*SL*_ value shows a significant drop. Once again, this drop is significantly higher at the positive potential compared to the negatively charged surface. To gain a better understanding of the ionic contribution to the observed trend in γ_*SL*_, we plot the water-only (calculated including merely the van der Waals and Coulomb interactions between water molecules and among water molecules and the electrode) and the ion-only (the difference between total γ_*SL*_ and the water contribution to the γ_*SL*_) contributions to γ_*SL*_ as a function of ionic concentration (Figure 4b,c). We find that in the electrolyte, the water contribution to γ_*SL*_ is similar for both negatively and positively charged surfaces, decreasing almost monotonically as the ionic concentration increases. This decrease is explained by the fact that as the concentration of electrolyte ions increases, the amount of water at the interface decreases (Figure S3e). At low concentration (1M), the surface tension is fully dominated by the contribution of water (i.e., the value of γ_*SL*_ in the solution is almost equal to that attributed exclusively to water; ionic γ_*SL*_ approaches zero), while at higher concentrations, the ionic contribution has a different effect depending on the type of ion (cations or anions) that is closer to the interface. Interestingly, ionic γ*_SL_* at the positively charged electrode is predicted to substantially decrease from 1 to 2 M followed by an increase at the highest concentration. A close look in the density profiles calculated for the positively charged surface in Figure S3 reveals that the thickness of the Helmholtz layer is different among the concentrations studied. In particular, as seen in Table S1, the OHP exhibits a minimum in a 2 M solution. Notably, this trend closely follows that observed in the dependence of ionic γ_SL_ on the electrolyte concentration (Figure 4c). A thicker Helmholtz layer suggests a more diffuse screening of the electrode charge in the immediate vicinity of the electrode, which is expected to upshift the value of ionic γ_SL_ (the increased presence of coions due to the overscreening effect dampens the repulsive interactions between Cl^–^ that weaken the surface tension.). Furthermore, the significantly higher density of ions inside the Helmholtz layer in the 4 M solution compared to those in 1 and 2 M (see the non-normalized density plots in Figure S3c) increases the pressure difference in eq 3, depicted in the positive shift of the ionic γ_*SL*_. A similar phenomenon is also seen at the negatively charged electrode, where a slight increase in the thickness of the OHP along with the higher density of ions in the Helmholtz plane in the most concentrated solution (see the non-normalized density plots in Figure S3b) shifts γ_*SL*_ to more positive values. Note that a direct comparison between the oppositely charged surfaces is not straightforward since the identity of the ions residing inside the Helmholtz layer is different. Overall, the above findings highlight the striking effect of the structure of the Helmholtz layer (ions and water molecules) on the interfacial surface tension. The predicted promotion of electrowetting driven by Cl^–^ anions at the positively charged surface is in agreement with the experimental work by Papaderakis et al.,^19^ who found that the adsorption of anions (F^–^) at positively charged surface enhances the electrowetting process. On the contrary, the presence of Li^+^ cations at the interface (i.e., at the negatively charged surface) results in a weaker effect on the γ_*SL*_ value (compared to the value at the neutral surface), this being particularly evident at the highest concentration of 4 M where the amount of Li^+^ accumulated at the interface is the highest (Figure S3b).

The results of Figure 4 indicate that there is a threshold concentration of 2 M, above which the change in γ_*SL*_ reaches a plateau. This conclusion is again in agreement with the work of Papaderakis et al.,^19^ which shows that electrowetting on HOPG increases as the ionic concentration (KF) increases, with a threshold of 5 M. The difference in the critical concentration values may be due to the use of different ionic species and electrode materials.

To explain the change in surface tension with ionic concentrations and surface charge, we calculate the difference between the cohesive energy, *E_coh_*, (calculated as the sum of water–water, water–ion, ion–ion van der Waals, and Coulomb energy contributions) and the adhesive energy, *E_adh_* (calculated as the sum of water–graphene and ion-graphene van der Waals and Coulomb energy contributions). Both *E_coh_* and *E_adh_* are calculated with a cutoff of 1.4 nm, which is the maximum permissible cutoff for our system size. The results presented in Figure 4d show that this difference decreases with the increase in ionic concentration while remaining negative, confirming that the presence of the ionic species makes the surface more hydrophilic.

### 3.3 Hydrogen Bond Numbers and Water Molecular Dipole Moment Angles

**Figure 5:**
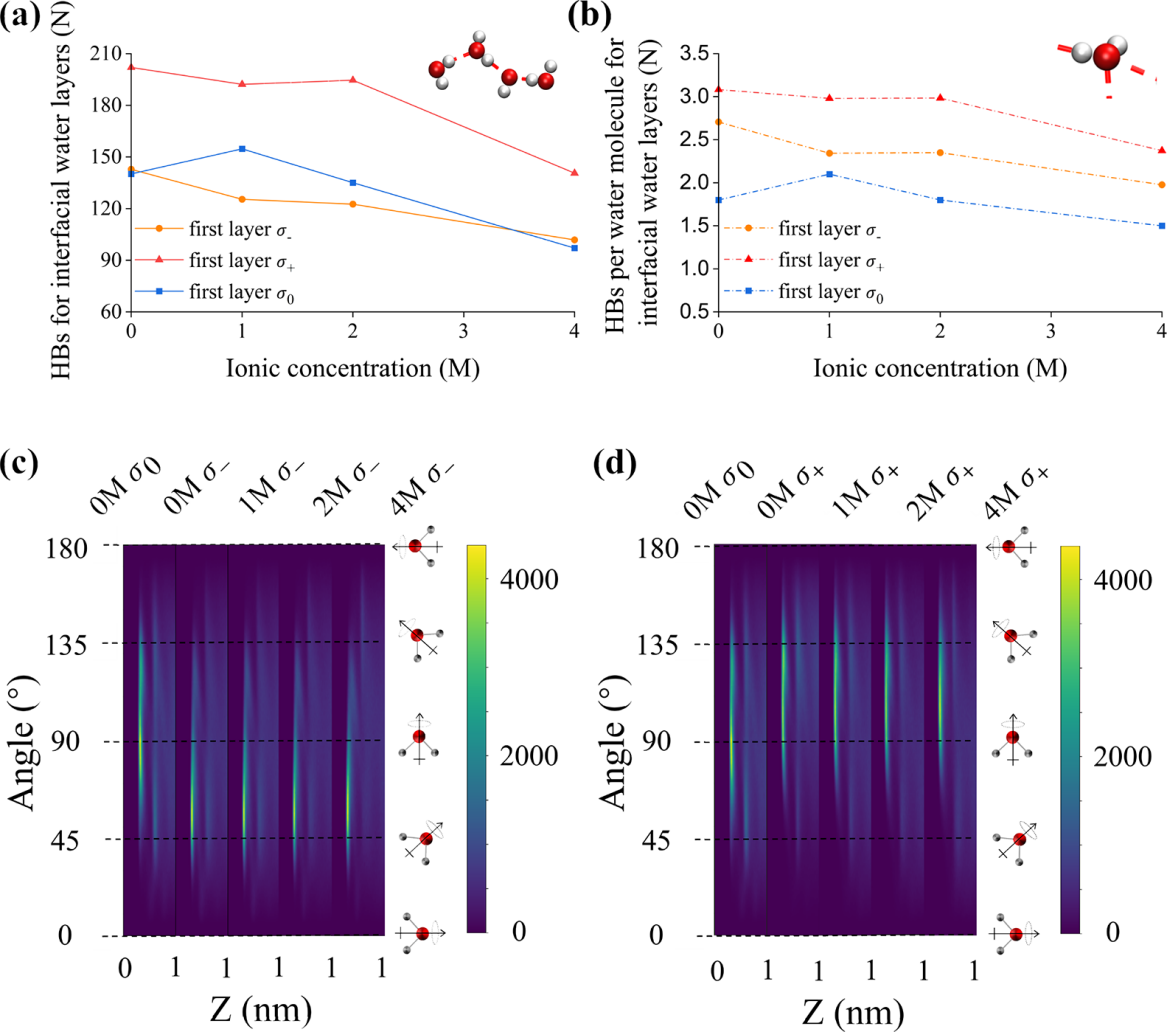
(a) Total number of water–water hydrogen bond calculated for the first interfacial water layers at different charged surfaces (σ_–_ and σ_+_) as a function of the ionic concentration. (b) As above but per water molecules. (c–d) Two-dimensional histogram reporting the angular orientation of the water molecular dipole moment relative to the neutrally and positively and negatively charged surface (σ_–_ and σ_+_) for different ionic concentrations (0, 1, 2, 4 M).

To investigate what drives the asymmetry of the electrowetting phenomenon, we calculate the numbers of interfacial water–water hydrogen bonds (HBs) (Figure 5a,b and Figure S5). We use the geometric criterion to determine whether a water molecule forms hydrogen bonds, *i.e*., with a distance between the oxygen atoms of the donor and the acceptor less than 0.35 nm and a corresponding H–O···O angle of less than 30°. The HBs are calculated only between water molecules that are included in the first and second layers relative to the interface. The boundaries of these layers are identified by using the density profiles reported in Figure 3.

Figure 5a and Figure S5a show that for the pure water systems, there is a clear correlation between the number of HBs and the surface tension: the interface with the lowest surface tension (water in contact with a positive surface) has the highest number of HBs. Wang et al.^65^ found a similar correlation for water wetting mica surfaces. This correlation is valid whether we look at the number of HBs in the first (Figure 5a) or second layer (Figure S5a). In line with the correlation identified for pure water, in the electrolyte solutions, the number of HBs for the interfacial water in contact with the positively charged surface (the most hydrophilic) is always higher than that calculated for the negatively charged and neutral surfaces, and it decreases with increasing ionic concentration. This correlation holds even if we account for the fact that the amount of interfacial water decreases with increasing salt concentration and calculate the number of hydrogen bonds (HBs) per water molecule Figure 5b. In general, we observe that for each ionic concentration, the number of HBs remains the highest for the positively charged surface, while the number of HBs at the negatively charged surface consistently exceeds that of the neutral surface.

The number of HBs in the second water layer is similar for negatively and positively charged surfaces at each concentration, indicating that the effect of the surface charge density on the structure of water in the second layer is negligible. Therefore, the structure of the first layer of water and ions seems to be the dominant factor governing the interfacial tension.

As expected, charging the surface affects the orientation of the interfacial water molecules, which is, however, not affected by the presence of the ions. In Figure 5c,d, we present the distribution of the water molecular dipole moment angles relative to the electrode surface as a function of the distance from the surface. An angle of 90° indicates parallel alignment, while 0° and 180° indicate vertical alignment with the water tail (hydrogen atoms) or water head (oxygen atom) pointing toward the surface respectively. In the pure water system, we observe that the water dipole moment in the first and second interfacial water layers tends to align parallel with the surface, with most water dipole angles in the first layer falling within the range of 60° to 135° and none are oriented perpendicular to the surface: this finding is consistent with previous studies.^30,66,67^ Upon charging the surface is charged and the ionic concentration increases, the water molecules in the first layer reorient themselves. On negatively charged surfaces, the angles range from 30° to 90°, while on positively charged surfaces, the range is from 80° to 150°. This means that the hydrogen/oxygen atoms in the first layer always face toward the negatively/positively charged surfaces, respectively. The results are consistent across concentrations.

Up to this point, we have demonstrated the asymmetric dependence of γ_SL_ on the applied surface charge of graphene in contact with pure water and aqueous solutions of varying LiCl concentrations. Variations in γ_SL_ were investigated within molecular distances from the graphene/solution interface and were interpreted based on the EDL structure, as that is governed by the interplay between electrostatic and intermolecular forces. It should be emphasized though that this combined “mechanical/thermodynamic” route relies on the definition of surface tension as originally formulated by Young in his equation^68^ and not the surface energy of the system. Hence, insights are provided into the force (tangential and normal) balance profiles extending from the first molecular layer adjacent to the electrode (the inner Helmholtz plane by electrochemical means), *i.e.*, immediately close to the contact line, to the interface of the diffuse layer and the bulk. As previously mentioned, the role of the electrode/solution interactions in the vicinity of the contact line and their influence on the free energy of the system has been studied by Taherian et al.,^28^ where the authors highlighted the much stronger influence of water molecular orientation on contact angle over that of γ_SL_ at relatively low charge densities (less than 2 μC cm^–2^). Here, the dominant role of γ_SL_ at higher charge densities is demonstrated. In fact, despite focusing our discussion on the integral value of γ_SL_ (*s*ee eq 3), variations in γ_SL_ with surface charge density and electrolyte concentration are detected already from the first molecular layer close to the electrode (Figure S4).

### 3.4 EDL Capacitance and Total Areal Capacitance

It is well-established that the electronic structure of graphene and in particular the finite number of densities of state (DOS) close to the Fermi level give rise to a voltage drop associated with the rate of change of the occupancy of the band edges in the electrode. The latter introduces a potential drop that is different from the Galvani potential, leading to an additional term to the EDL capacitive contribution added in series with the latter, typically referred to as the quantum capacitance (*C*_*Q*_).^69^ On this basis, the total capacitance, *C*, of the system is expressed as the series contributions of *C*_*Q*_ and the EDL capacitance (*C*_*EDL*_), as follows:
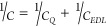
6

Thus, the *C*_*Q*_ contribution originates from the electronic structure on the electrode,^70^ which, for graphene, is usually limited by its low electronic density of states near the Fermi level^71^ and can be calculated using DFT and DFTB;^43^*C*_*EDL*_ represents instead the contribution of the charge accumulation within the EDL at the electrode/electrolyte interface and can be calculated using MD simulations using the difference of the electrostatic potential drop (ΔΦ) between the charged and neutral charged graphene case, ΔΔΦ, as follows:
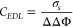
7where σ_*s*_ is the surface charge density.

To calculate this ΔΔΦ, we first extract the charge density, ρ(*z*), from the number density profile along the *z* direction, *n*(*z*). This is achieved by multiplying the atom-resolved number densities by their respective partial charges. The ρ(*z*) includes charged atoms in water, ions, and charged graphene. The curvature of the electrostatic potential, Φ, is connected to ρ(*z*) as dictated by Poisson’s equation:^31^
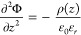
8where ε_0_ and ε_*r*_ are the vacuum permittivity constant and specific electrolyte permittivity, respectively. We obtain Φ(*z*) by integrating eq 8 from the bulk layer (the net charge density is zero) to the surface of the charged graphene.

The electrostatic potential of the bulk layer, due to its net electroneutrality, serves as our reference point and is denoted as Φ^*ref*^. This neutrality ensures a consistent electrostatic potential value. The subsequent electrostatic potential drop across the interface is expressed as ΔΦ = Φ^*electrode*^ – Φ^*ref*^,^31^ with Φ^*electrode*^ being the electrostatic potential at the charged graphene surface. Ultimately, ΔΔΦ is defined as ΔΦ – ΔΦ^0^, where ΔΦ^0^ corresponds to the electrostatic potential drop associated with the neutrally charged graphene case.^31^ All Φ, ΔΦ, and ΔΔΦ values are shown in Figure S6. Electrowetting mainly influences *C*_*EDL*_ by changing the structure of EDL^2^.

To investigate whether the change in γ_*SL*_ is mirrored by a change on the mechanism of charge storage at the interface as predicted by the Lippmann equation, we calculate the predicted total areal capacitance (*C*_*YL*_) using eq 2 and compare it with the total areal capacitance (*C*) calculated using eq 6. The value of *C*_*EDL*_ is determined using eq 7, and the value of *C*_*Q*_ is taken elsewhere,^31,43^ where the same molecular model and surface charge was used. These values for both negatively and positively charged surfaces are presented in Table S3. As shown before,^31^ the ΔΔΦ values remained relatively constant despite varying ionic concentrations, resulting in a negligible change in *C*_*EDL*_. This suggests that the increase in the number of ions in the first adsorption layer has a limited effect on *C*_*EDL*_, but it does result in a reduction in the value of γ_*SL*_. All values of *C* across various ionic concentrations (less than 5 μF cm^–2^) lie within the range of those reported for aqueous LiCl solutions on HOPG (less than 8 μF cm^–2^ for positive potentials depending on electrolyte concentration) at similar surface charges (*i.e.*, ca. +1.3 V relative to the potential of the neutral surface).^56^ The differences most probably arise from the decreased number of DOS near the Fermi level in graphene and the pseudocapacitive contributions (*i.e*., adsorption of Cl^–^) on HOPG at increased positive potentials.

**Figure 6:**
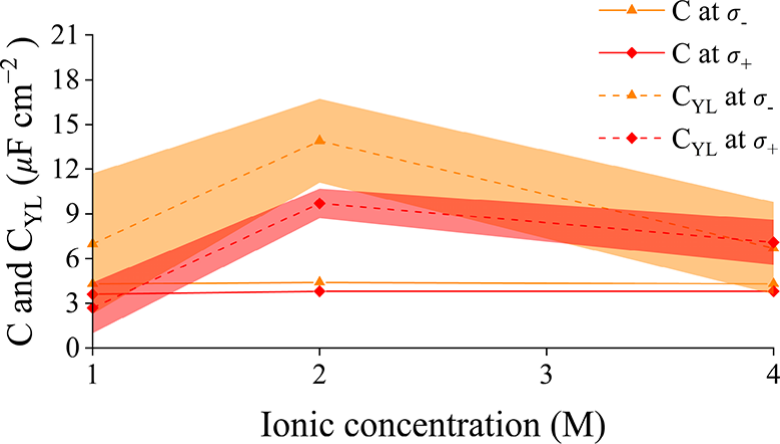
Comparison between the capacitance calculated from QM/MD simulations, *C*, and that calculated using eq 2 and the calculated value of *Y*_*SL*_, *C_YL_* with standard deviation (shaded error bands).

The comparison of the capacitance values, *C*, with those obtained using eq 2 commonly used to predict total areal capacitance at the macroscopic level, is revealing (Figure 6). While *C*_*YL*_ and *C* are within the error at both negative and positive charged surfaces for 1 and 4 M LiCl solutions, the Young–Lippmann equation predicts capacitance values consistently larger than those calculated from the simulations. This becomes even more evident for the 2 M LiCl solution, where the value of *C*_*YL*_ is significantly larger than that of *C*. This is due to a significant change in γ_*SL*_, which results in a large increase in *C*_*YL*_ in the 2 M solution. Notably, it can be seen from the tabulated data that *C* is larger at the negatively biased electrode compared with the positively charged surface. This apparent counterintuitive finding (considering the opposite trend in the calculated γ_*SL*_ values, Table S2) can be interpreted based on the density profiles discussed in Section 3.1 (see Figure 3 and Figure S3). Overscreening occurring at the positively biased surface results in the formation of alternating/overlapping ion and counterion layers (as is evident in the coexistence of Cl^–^ and Li^+^ at positive bias; see Figure 3 and Figure S3) that leads to a more diffuse screening of the positively charged surface compared to the negatively biased electrode. This “asymmetric” accumulation of ions in the EDL at opposite signs of surface charge is responsible for the higher *C* values in the negatively biased electrode. In addition, it is also worth highlighting recent molecular dynamics calculations of the nonlinear response of the effective permittivity of H_2_O in contact with oppositely charged graphene electrodes to applied electric fields.^72^ At bias comparable to the present work, it has been observed that not only is there an asymmetry in the effective permittivity due to the reorientation of the solvent molecules with the field, the ratio of the permittivity at the negative vs positive electrode is 2. Similarly, the computed capacitance (*C*_YL_) values for our lowest electrolyte concentration (1M), where solvent (H_2_O) effects are expected to be most dominant, also has a ratio of approximately 2, as can be seen in Figure 6. From the observed asymmetry in *C*, two important conclusions can be drawn: (i) in line with previous studies,^43,73^ graphene is able to store a higher amount of charge under negative bias; and (ii) the fact that γ_*SL*_ is not governed solely by the charge stored at the interface as the Young–Lippmann equation postulates but is also strongly dependent on the structure of the solvent in the inner and outer Helmholtz plane arising from the electrode/solvent specific interactions (see also the discussion in Section 3.3).

### 3.5 Experimental Investigation of Electrowetting on HOPG

**Figure 7:**
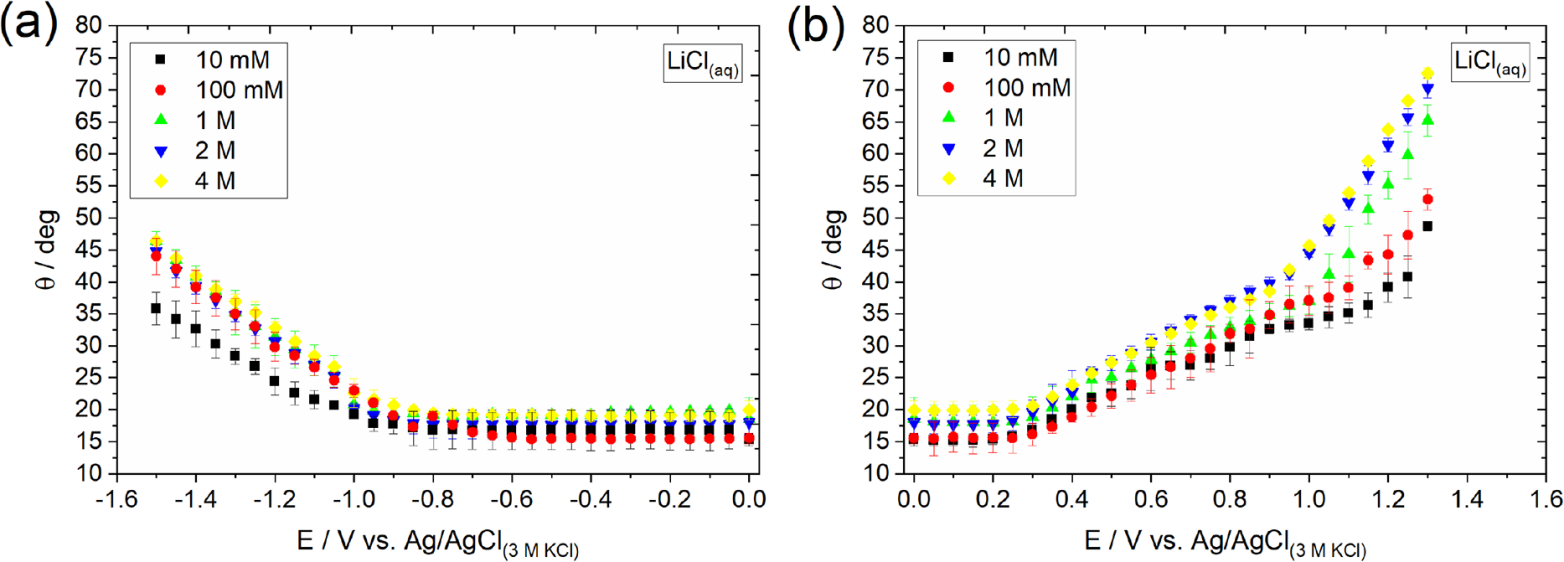
Electrowetting curves recorded following the experimental procedure described in Methods on a freshly cleaved HOPG electrode in contact with an insulating (no electrolyte added) DCB droplet immersed in aqueous LiCl solutions of varied concentration. The experimental setup and the mechanism of the process are illustrated in Figure 2 and described in detail in Methods. Overall, a decrease in γ*_SL_* at the HOPG/LiCl interface results in an increase of the DCB droplet’s contact angle.

The surface tension results obtained from the QM/MD simulations are now compared to the electrowetting response recorded experimentally on the basal plane of HOPG. Figure 7 presents the electrowetting response of HOPG recorded over a wide potential range in aqueous LiCl solutions of varied concentrations, following the experimental procedure described in Methods. The first noteworthy outcome is the observed asymmetry relative to the potential of zero charge (*E*_*pzc*_) in the dependence of the contact angle on the applied potential bias. *E*_*pzc*_ is reported to be ca. +0.1 V vs Ag/AgCl_(3.5 M KCl)_ for concentrations up to 1 M LiCl.^56^ Notably, at potentials more positive than *E*_*pzc*_ (Figure 7b), an increase in electrolyte concentration results in an increase of the insulating droplet’s contact angle. As described in the Methods and illustrated in Figure 2b, this phenomenon is driven by the decrease in the interfacial surface tension, γ_*SL*_, between the electrode and the electrolyte. The higher the extent of the decrease in γ_*SL*_, the more pronounced its effect on the contact angle of the insulating (no electrolyte) droplet; *i.e.*, larger contact angle values are expected. In particular, at +1.3 V, corresponding roughly to the charge density used in the simulations, the recorded contact angle increases from ca. 49° for a 10 mM solution to ca. 70° for 2 M, demonstrating the decrease in γ_*SL*_ with LiCl concentration. Above 2 M, the observed response remains almost unaffected (only a slight increase by 3° is seen), indicating a plateau for the effect of the electrolyte concentration on electrowetting. Both the experimentally observed asymmetry in the electrowetting curves and the decrease in γ_*SL*_ with electrolyte concentration at a positively polarized surface with a concentration plateau at 2 M are in complete agreement with our simulation results (see section 3.2). However, we should stress the nonideally polarizable character of the interface during the experiments at applied potentials more positive than ca. +0.7 V. As previously reported, faradaic processes involving the specific adsorption of Cl^–^ ions occur at biases more positive than +0.7 V and the rate of the reaction increases with LiCl concentration.^56^ These effects are expected to influence electrowetting by decreasing γ_*SL*_;^74,75^ thus, they cannot be compared with the simulation results where reactions between the electrode and electrolytes cannot be modeled without fully ab initio molecular dynamics.

At potentials more negative than *E*_*pzc*_ (Figure 7a), the experimental electrowetting responses show no dependence on electrolyte concentration, except for the lowest concentration, *i.e.*, 10 mM. This is consistent with our simulation results for the 2 and 4 M LiCl, but not for the 1 M. The apparent discrepancy between the experimental data and the model for 1 M might be related to electrode-specific interactions with the solvent inside the IHP (*e.g.*, higher number of HBs) that are too subtle to be picked up by the contact angle measurements as well as the occurrence of underlying electrochemical reactions that have not been considered in the QM/MD model. The latter have been shown to have a stronger impact on the electrowetting response at lower electrolyte concentrations.^19^

## 4 Conclusions

In summary, we have used QM/MD simulations to investigate the electrowetting phenomena occurring at the graphene/electrolyte interface. In line with previous experiments and Young–Lippmann theory, the simulations indicate that graphene surfaces become more hydrophilic upon charging and that the presence of the ions enhances this effect, reducing the surface tension even further. Contrary to the prediction of the YL theory and as shown by some previous simulations of the contact angle,^28^ we observe that electrowetting is an asymmetric phenomenon and that different levels of wetting occur at different signs of applied voltage, with negative surfaces being systematically more hydrophobic than positive ones. We observe that the effect of the ionic concentration on the surface wettability is not trivial and that it saturates for concentrations larger than 2 M. Experiments carried out on HOPG confirm our simulation data and are in excellent agreement with the computational predictions. The rationalization of the surface tension results proved to be difficult. Competitive effects, such as ion adsorption and hydrogen bond number, contribute to the surface tension values. Overall, we notice a correlation between the number of interfacial water–water hydrogen bonds and the hydrophilicity of the surface. Finally, we compare the electrochemical capacitance with that predicted by the Young–Lipmann equation. We found that while the individual values lay within the simulation uncertainty for two out of the three concentrations investigated here, the trend of the predicted capacitance as a function of ionic concentration is wrong, indicating that the EDL structure (related to the electrochemical capacitance) is not directly correlated with the wettability of the surface and different interfacial mechanisms drive the two phenomena.

## Supporting Information Available

QM/MD framework/methodology, number density plots of hydrogen and oxygen in pure water, non-normalized density profiles of electrolyte ions and water molecules, normal and tangential pressure difference with the running integral value of interfacial tension along z direction, hydrogen bonds in the second interfacial water layer, electrostatic potential profiles and the electrostatic potential drop at the charged and neutral surfaces, tabulated values of the inner and outer Helmholtz planes as well as the electrode/electrolyte surface tension, electrostatic potential drop and capacitance (PDF)
