# Supplementary material for: Relation between Double Layer Structure, Capacitance, and Surface Tension in Electrowetting of Graphene and Aqueous Electrolytes
Source: J Am Chem Soc. 2023 Dec 28;146(1):760–72. doi: 10.1021/jacs.3c10814 (PMC10785801; doi:10.1021/jacs.3c10814)
Supplement: Supplementary file 1 — ja3c10814_si_001.pdf [file ja3c10814_si_001.pdf]

Relation between double layer structure, capacitance and surface tension in electrowetting of graphene and aqueous electrolytes

Zixuan Wei[1], Joshua D. Elliott[2], Athanasios A. Papaderakis[3], Robert A.W. Dryfe[3*], Paola Carbone[1*]*

[1]Department of Chemical Engineering, The University of Manchester, Oxford Road, M13 9PL, Manchester (UK)

[2]Diamond Light Source, Diamond House, Harwell Science and Innovation Park, Didcot, Oxfordshire, OX11 ODE, United Kingdom

[3]Department of Chemistry and Henry Royce Institute, The University of Manchester, Oxford Road, M13 9PL, Manchester (UK)

Supporting Information

∗To whom correspondence should be addressed. Email: paola.carbone@manchester.ac.uk; robert.dryfe@manchester.ac.uk; athanasios.papaderakis@manchester.ac.uk

Contents

List of Figures

Figure S1. Schematic representation of the QM/MD framework with post-processing to calculate γ_{SL}. Blue and green dashed boxes represent main and sub iterative processes, respectively. S4

Figure S2. Oxygen (OW) and hydrogen (HW) number density of pure water calculated along the direction z, perpendicular to the surface: (a) neutral electrode (σ_0). (b) negative and positively charged electrodes (σ_- at the left side and σ_+ at the right side)... S5

Figure S3. (a) Li cation and Cl anion number densities at the neutral electrode (σ_0) and (b) at the negatively charged electrode (σ_-) and (c) at positively charged electrode (σ_+) as the function of the ionic concentration. (d) Water number density at the neutral electrode (σ_0) and (e) at the negatively charged electrode (σ_-) and (f) at the positively charged electrode (σ_+) as the function of the ionic concentration. S6

Figure S4. (a-f) Normal and tangential pressure difference $PN(z) - PT(z)$ with the running integral value of γ_{SL} along z of the system (water with ions) with different charged surfaces and ionic concentration. (g-j) That of the pure water system. (k-n) That of the pure ion system. S9

Figure S5 (a) Total number of water-water hydrogen bond calculated for the second interfacial water layers at different charged surfaces (σ_- and σ_+) as the function of the ionic concentration. (b) As above, but per water molecule... S10

Figure S6. Electrostatic potential, Φ (V) and the difference of electrostatic potential drop at the charged and neutral surfaces, ΔΦ (V) and ΔΔΦ (V), as functions of ionic concentration.............................. S12

List of Tables

Table S1. The thickness of inner and outer Helmholtz planes (IHP and OHP) at different charged surfaces (σ_- and σ_+) with different ionic concentrations.. S13

Table S2. The γSL at different charged surfaces (σ_0, σ_- and σ_+) with different ionic concentrations. S14

Table S3. Electrostatic potential drop difference, ΔΔΦ (V) and capacitance values (μF cm^{-2}) for different ionic concentrations. S15

1. Methodology: the QM/MD workflow

The QM/MD simulation is based on an innovative iterative process. This process combines quantum mechanical calculations of the electrode's electronic structure using the self-consistent charge (SCC) density functional Tight-Binding (DFTB), with classical molecular dynamics (MD) simulations of the electrolyte. The goal of this approach is to understand the dynamics and structural changes at the electrode/electrolyte interface during electrolyte-induced polarization.

The QM/MD framework consists of four steps:

(i) Converting the electrolyte atoms into point charges based on their Cartesian coordinates and partial charges in the force field for a given time, t.

(ii) Calculating the electronic structure of the electrode using the point charges as the background electrostatic potential.

(iii) Post-processing the electronic structure to obtain a set of atomic charges.

(iv) Updating the electrode atomic charges into the classical force field and using MD simulations to obtain a short dynamical trajectory.

This iterative process continues to drive the classical dynamics, and there is a sub-loop of post-processing to obtain the γ_{SL}. The overall QM/MD framework, including the post-processing, is illustrated in **Figure S1**.

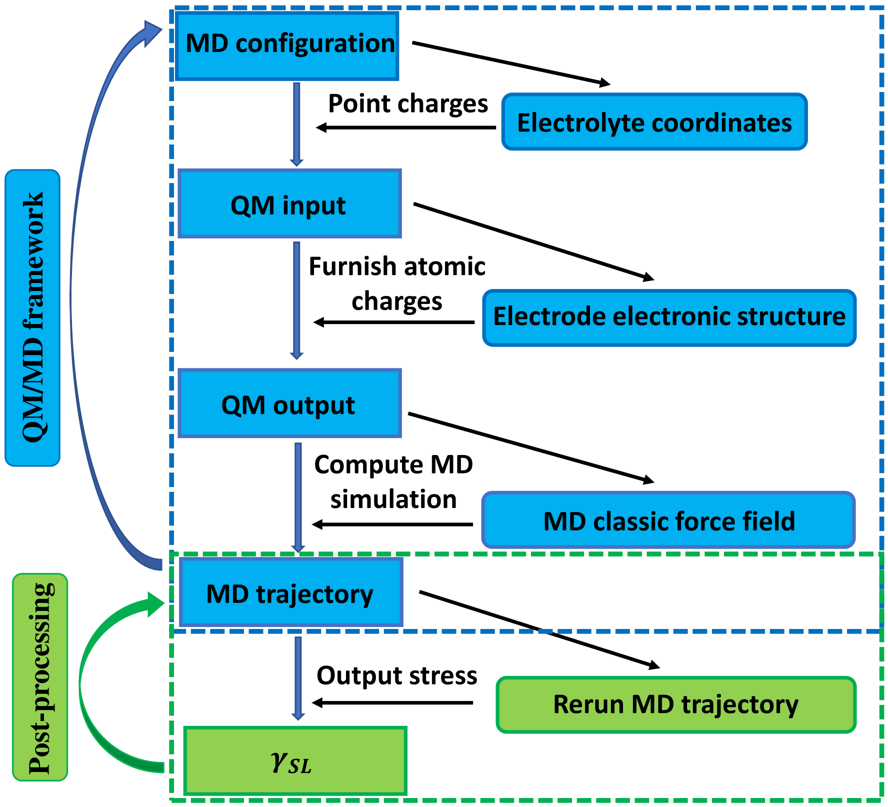

Figure S1. Schematic representation of the QM/MD framework with post-processing to calculate γ_{SL}. Blue and green dashed boxes represent main and sub iterative processes, respectively.

2. Number density of oxygen and hydrogen

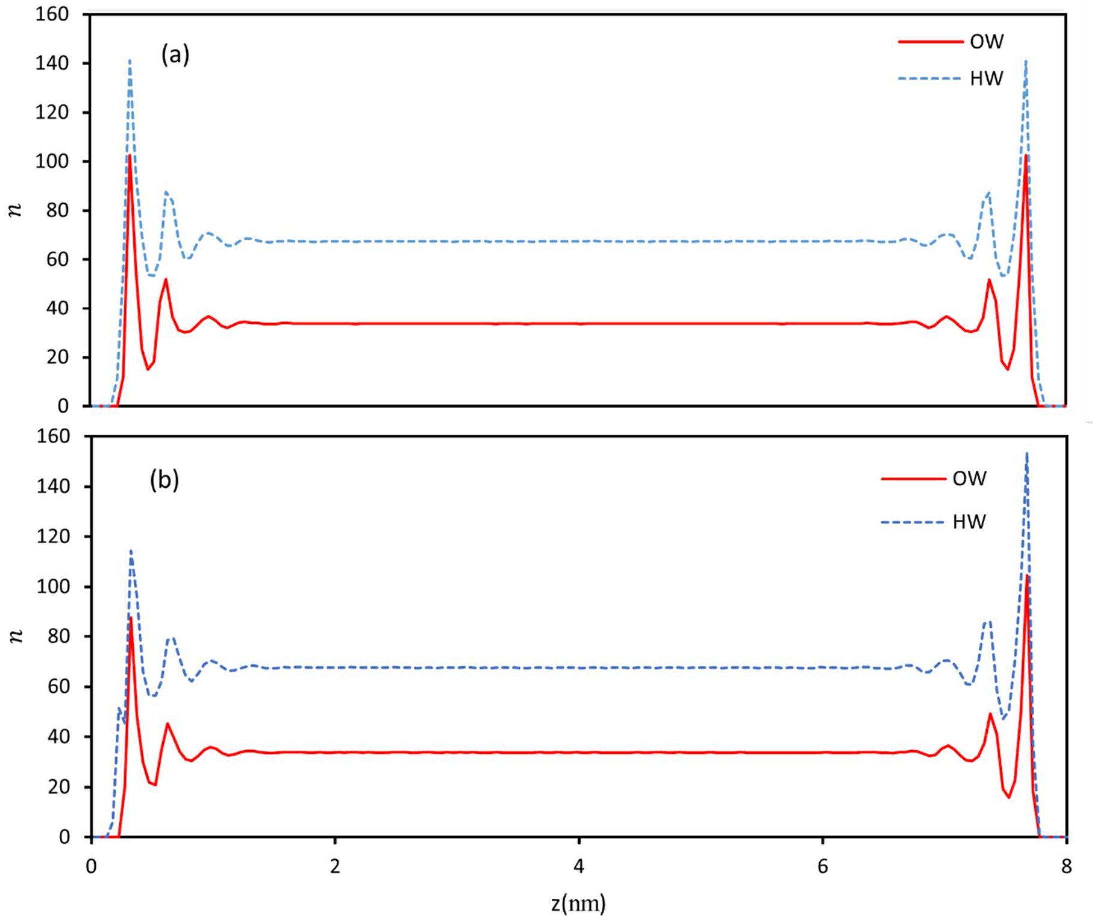

Figure S2. Oxygen (OW) and hydrogen (HW) number density of pure water calculated along the direction z, perpendicular to the surface: (a) neutral electrode (σ_0). (b) negative and positively charged electrodes (σ_- at the left side and σ_+ at the right side).

3. Number density of Li cations and Cl anions

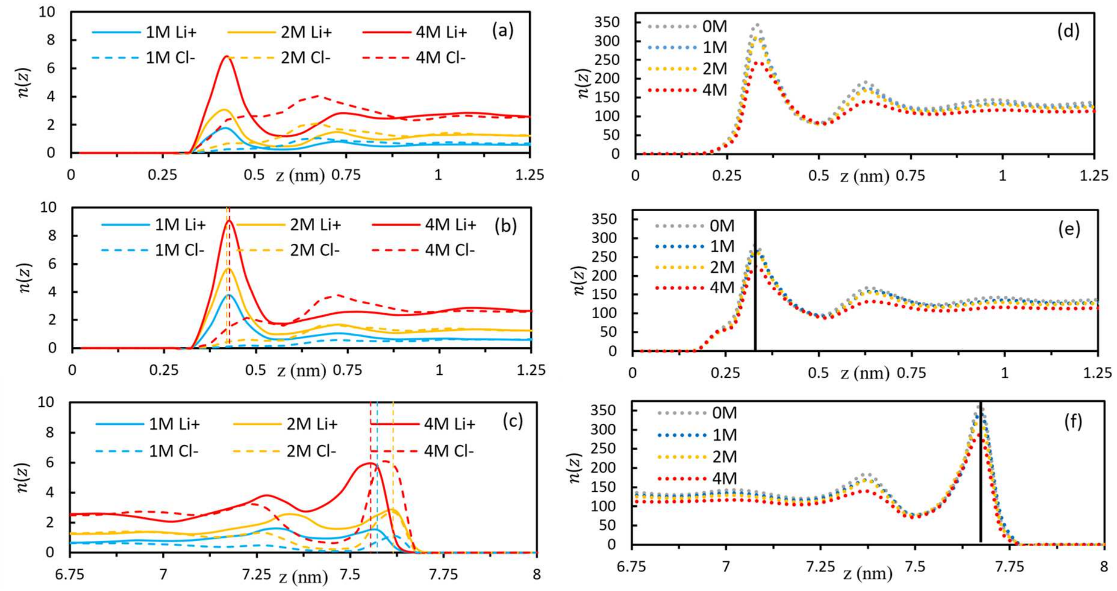

Figure S3. (a) Li cation and Cl anion number densities at the neutral electrode (σ_0) and (b) at the negatively charged electrode (σ_-) and (c) at positively charged electrode (σ_+) as the function of the ionic concentration. (d) Water number density at the neutral electrode (σ_0) and (e) at the negatively charged electrode (σ_-) and (f) at the positively charged electrode (σ_+) as the function of the ionic concentration. All vertical dashed lines in (b) and (c) represent OHP with different colours relating to the different concentrations. All vertical black lines in (e) and (f) represent to IHP.

4. Normal and tangential pressure difference with the running integral value of interfacial tension along *z* direction.

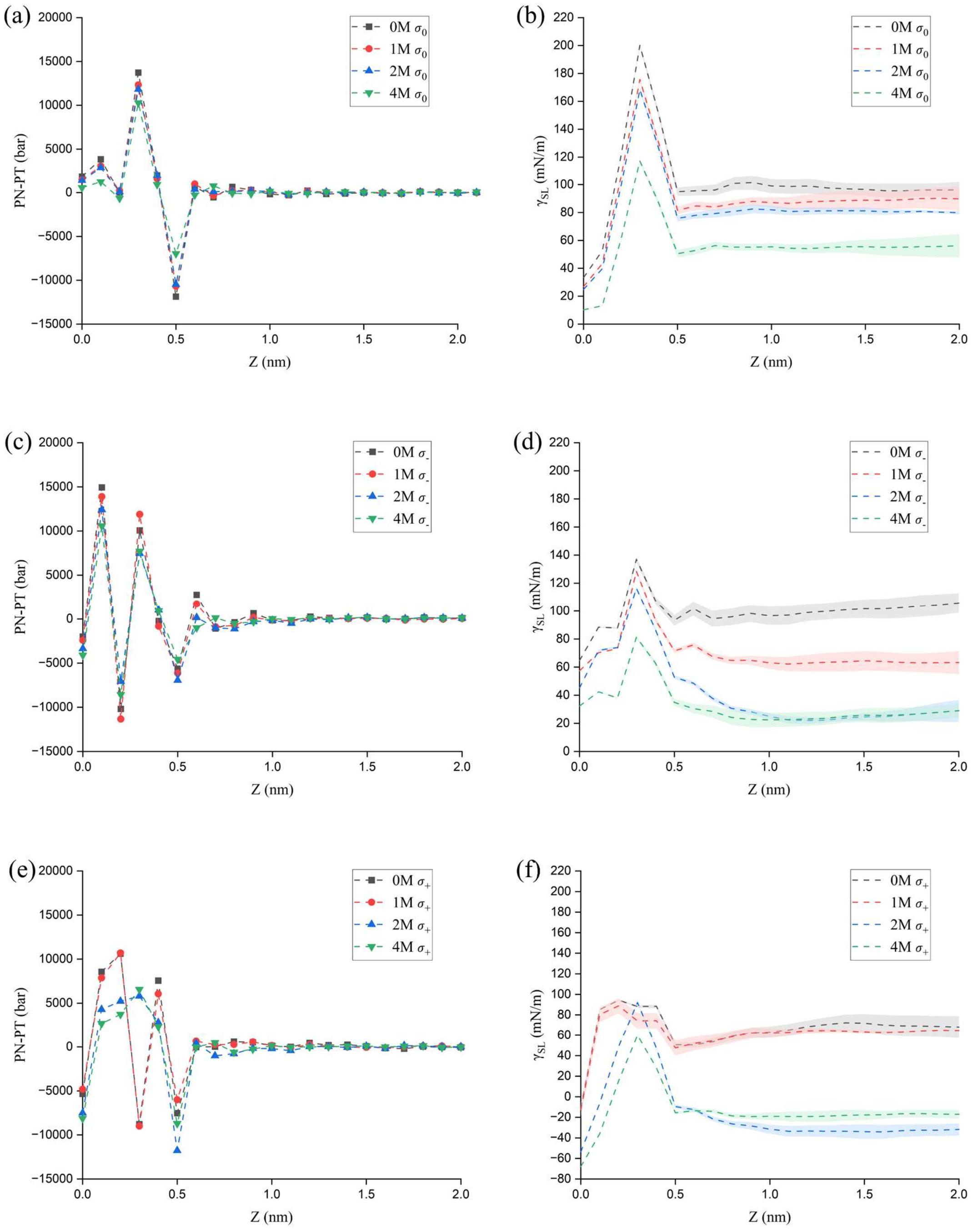

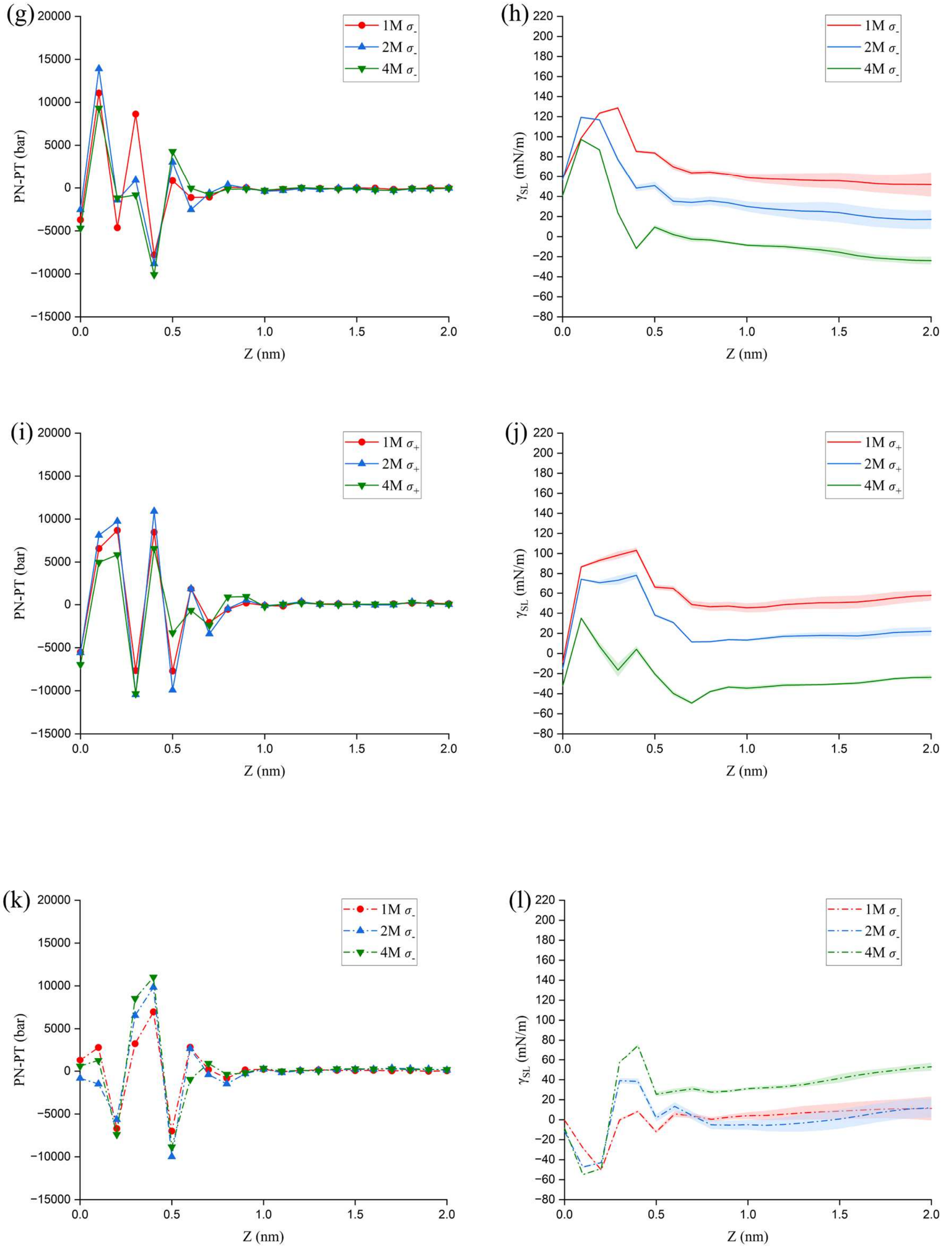

(g)
PN-PT (bar)
Z (nm)
1M σ_-
2M σ_-
4M σ_-
(h)
γ_{SL} (mN/m)
Z (nm)
1M σ_-
2M σ_-
4M σ_-
(i)
PN-PT (bar)
Z (nm)
1M σ_+
2M σ_+
4M σ_+
(j)
γ_{SL} (mN/m)
Z (nm)
1M σ_+
2M σ_+
4M σ_+
(k)
PN-PT (bar)
Z (nm)
1M σ_-
2M σ_-
4M σ_-
(l)
γ_{SL} (mN/m)
Z (nm)
1M σ_-
2M σ_-
4M σ_-

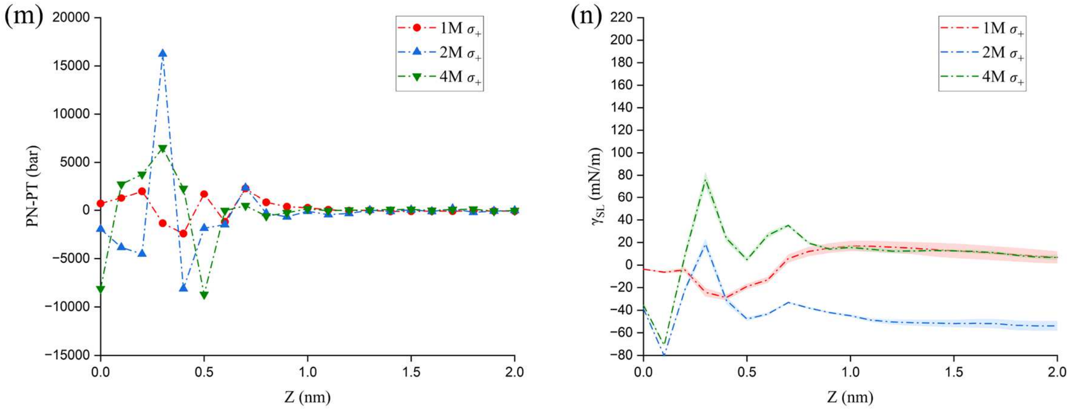

Figure S4. (a-f) Normal and tangential pressure difference $P_N(z) - P_T(z)$ with the running integral value of γ_{SL} along z of the system (water with ions) with different charged surfaces and ionic concentration. (g-j) That of the only water contribution. (k-n) That of the only ionic contribution.

5. HBs of second interfacial water layer

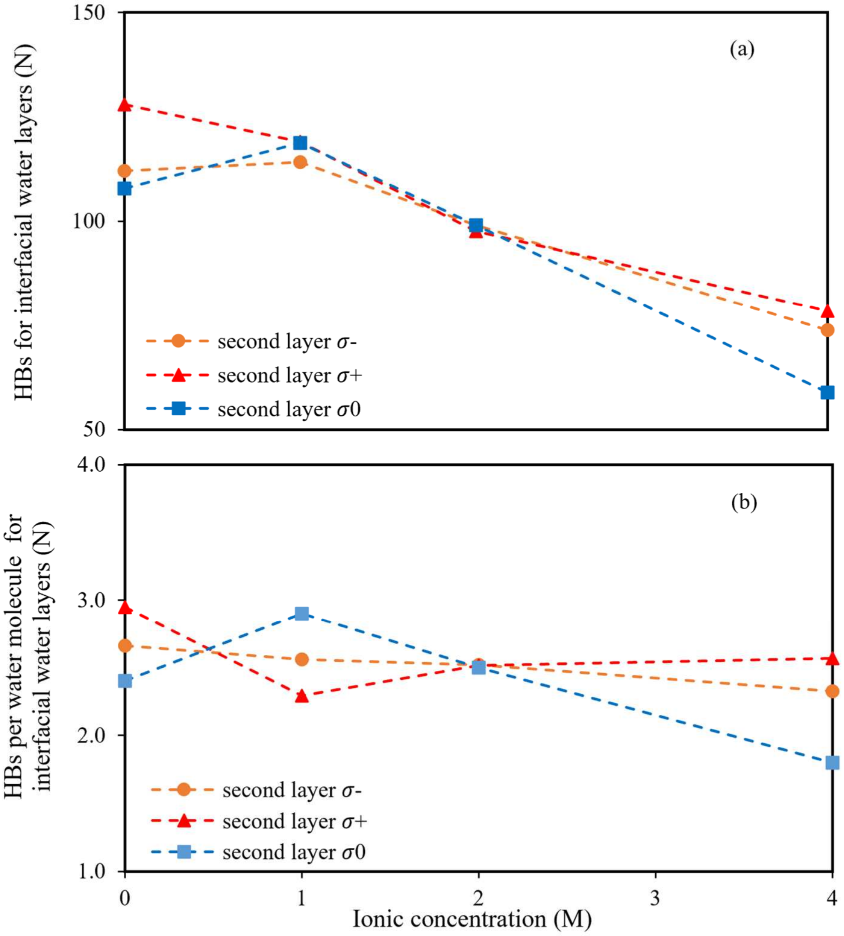

***Figure S5** (a) Total number of water-water hydrogen bond calculated for the second interfacial water layers at different charged surfaces (σ_- and σ_+) as the function of the ionic concentration. (b) As above, but per water molecule.*

6. Electrostatic potential and the difference of electrostatic potential drop

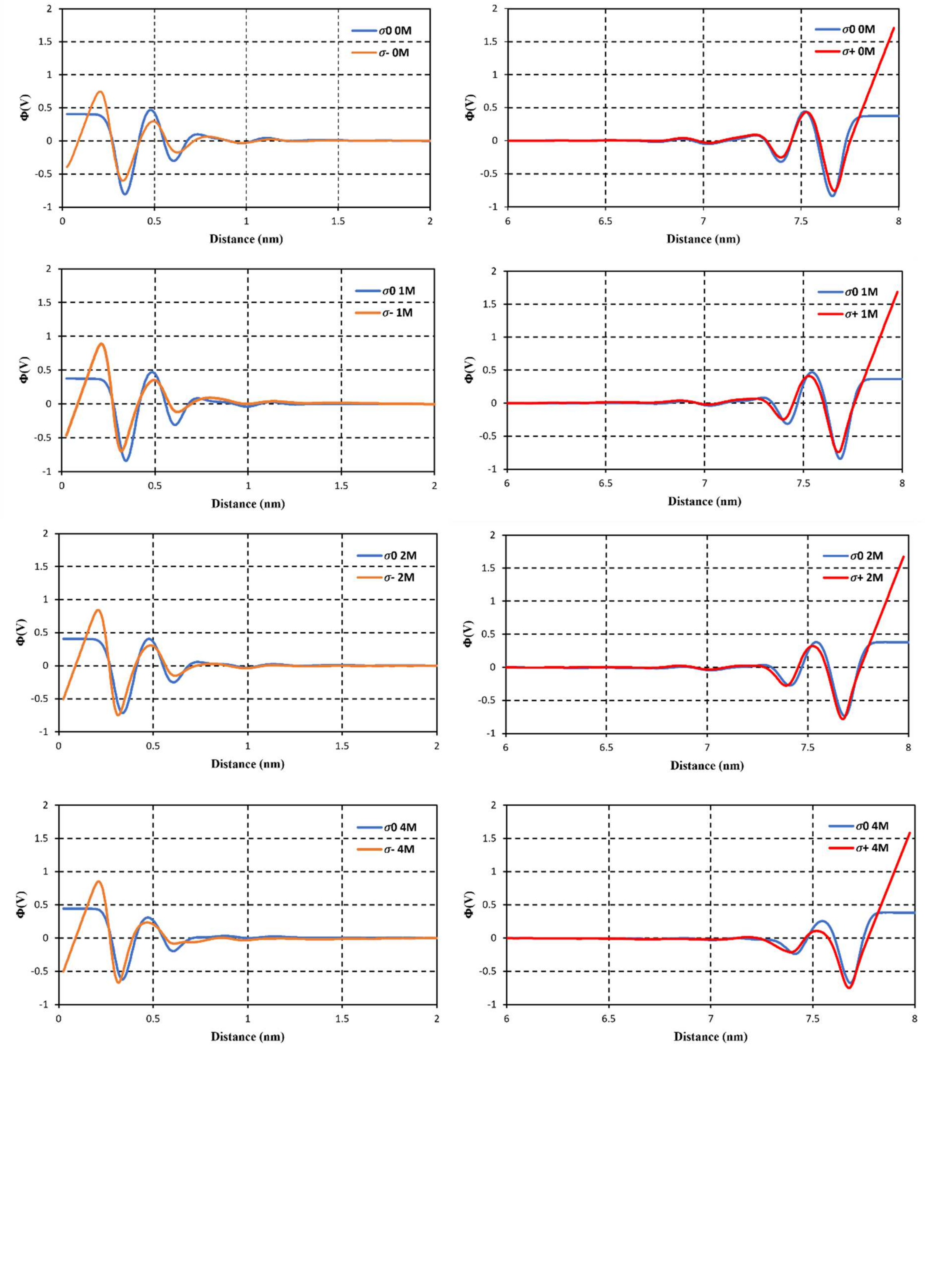

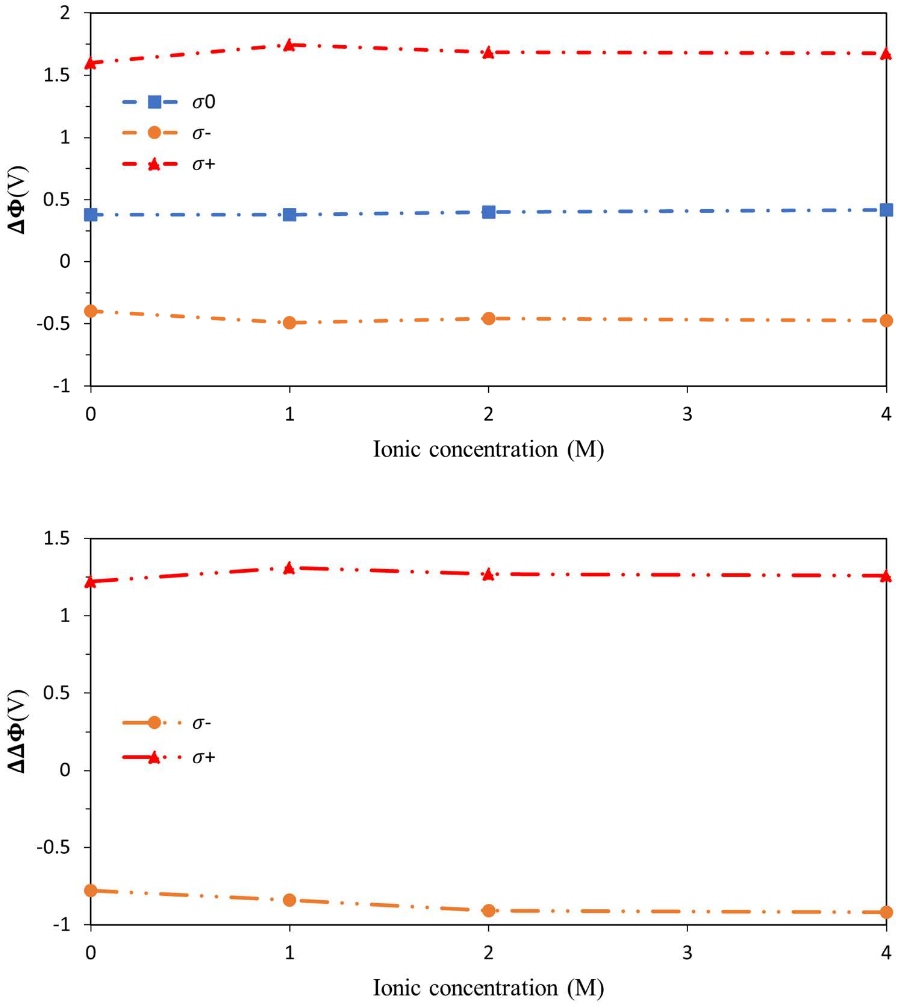

Figure S6. Electrostatic potential, Φ (V) and the difference of electrostatic potential drop at the charged and neutral surfaces, ΔΦ (V) and ΔΔΦ (V), as functions of ionic concentration.

7. The thickness of inner and outer Helmholtz planes

Table S1. The thickness of inner and outer Helmholtz planes (IHP and OHP) at different charged surfaces (σ_- and σ_+) with different ionic concentrations.

**Ionic concentration (M)**	**Thickness of IHP at σ_- (nm)**	**Thickness of IHP at σ_+ (nm)**	**Thickness of OHP at σ_- (nm)**	**Thickness of OHP at σ_+ (nm)**
**1**	0.33	0.33	0.42	0.43
**2**	0.33	0.33	0.42	0.39
**4**	0.33	0.33	0.43	0.45

8. The electrode/ electrolyte interfacial tension

Table S2. The γ_{SL} at different charged surfaces (σ_0, σ_- and σ_+) with different ionic concentrations.

Ionic concentration (M)	γ_{SL} at σ_0 (mN/m)	γ_{SL} at σ_- (mN/m)	γ_{SL} at σ_+ (mN/m)
**0**	96.2 ± 7.3	105.8 ± 7.0	68.1 ± 10.7
**1**	89.8 ± 9.5	63.2 ± 8.2	64.7 ± 6.1
**2**	79.9 ± 2.4	28.9 ± 7.8	-31.8 ± 5.9
**4**	56.1 ± 7.6	29.1 ± 4.9	-17.0 ± 4.1

9. The electrostatic potential drop difference and capacitance

Table S3. Electrostatic potential drop difference, $\Delta\Delta\Phi$ (V) and capacitance values ($\mu F\ cm^{-2}$) for different ionic concentrations.

	Negative charged surface σ_-					Positive charged surface σ_+				
**Ionic concentration (M)**	$\Delta\Delta\Phi$	C_{EDL}	C_Q	C	C_{YL}	$\Delta\Delta\Phi$	C_{EDL}	C_Q	C	C_{YL}
**1**	-0.87	8.2	9.1	4.3	7.0±4.7	1.36	5.2	12.1	3.6	2.7±1.7
**2**	-0.86	8.3	9.4	4.4	13.9±2.8	1.29	5.5	11.8	3.8	9.7±1.0
**4**	-0.9	7.9	9.2	4.3	6.7±3.1	1.26	5.6	11.5	3.8	7.1±1.5
